# Effect of Exposure to Famine during Early Life on Risk of Metabolic Syndrome in Adulthood: A Meta-Analysis

**DOI:** 10.1155/2020/3251275

**Published:** 2020-03-05

**Authors:** Lu-Lu Qin, Bang-An Luo, Fan Gao, Xiang-Lin Feng, Jia-He Liu

**Affiliations:** ^1^Key Laboratory of Molecular Epidemiology of Hunan Province, School of Medicine, Hunan Normal University, Changsha 410081, China; ^2^Department of Social Medicine and Health Management, School of Medicine, Hunan Normal University, Changsha 410081, China; ^3^Department of Mental Health, Brain Hospital of Hunan Province, Changsha, 410007 Hunan, China; ^4^Department of Health Monitoring, Xi'an Center for Disease Control And Prevention, Xi'an, Shaanxi 710054, China

## Abstract

**Background:**

Emerging studies have explored the association between the famine exposure during early life and the risk of the metabolic syndrome, and the results remain controversial. This meta-analysis was performed to summarize the famine effects on the prevalence of metabolic syndrome (MetS) in adulthood. *Materials and Methods.* We searched the PubMed, Web of Science, Embase, ScienceDirect, and Chinese National Knowledge Infrastructure for relevant studies up to December 2019. Pooled odd ratios (ORs) with 95% confidence intervals (CIs) were used to estimate the effect exposure to famine on MetS using a random-effects model, and the *I*^2^ was used to evaluate the heterogeneity.

**Results:**

The analyses included 39 studies from 10 articles with a total of 81504 participants. Fourteen studies from 10 articles for fetal famine exposure, 20 studies from 7 articles for childhood famine exposure, and 5 studies from 3 articles for adolescence/adult famine exposure were included in this meta-analysis. Compared with a nonexposed group, famine exposure significantly increased the risk of MetS for early life famine exposure (OR = 1.27, 95% CI: 1.18-1.38), fetal famine exposure (OR = 1.27, 95% CI: 1.14-1.43), and childhood famine exposure (OR = 1.29, 95% CI: 1.16-1.44). Subgroup analyses showed that the result was consistent regardless of the study designs, definitions of MetS, and causes of famine, with or without adjustment for age, smoking, drinking, and physical activity.

**Conclusions:**

This meta-analysis suggests that exposure to famine during early life may increase the risk of MetS in adulthood.

## 1. Introduction

Metabolic syndrome (MetS) refers to the pathological state that the metabolic abnormalities gathered and mutually connected, such as hypertension, hyperglycemia, elevated triglycerides, low high-density lipoprotein cholesterol, and central obesity, and these metabolic derangements increase the risk of type 2 diabetes mellitus, cardiovascular disease, chronic kidney disease, some cancers, and all-cause mortality among the adult population [1, 2]. Recently, sleep apnea, nonalcoholic fatty liver disease, chronic proinflammatory, and prothrombotic states have been added to the components of MetS, making the definition of MetS more complex [3]. However, there is still no universally accepted pathogenic mechanism and clearly defined diagnostic criteria [4].

Studies have demonstrated that the prevalence of MetS is increasing worldwide. According to the National Health and Nutrition Examination Survey (NHANES) 2003-2012, the prevalence of MetS in the United States had increased from 32.9% in 2003-2004 to 34.7% in 2011-2012 [5]. And the Korea National Health and Nutritional Examination Survey (KHANES) showed that the prevalence of MetS in Korea had increased from 24.9% to 31.3% between 1998 and 2007 [6]. Genetic susceptibility, smoking, drinking, and the lack of physical activity are widely recognized as important risk factors for MetS [7–11]. In addition, research studies have indicated that undernutrition during early life results in increasing risks of type 2 diabetes, hypertension, and obesity [12–14], all of which are the components of MetS. And relevant studies also confirmed these conclusions [15–17].

In recent years, a few studies have been performed to investigate the relationship between famine exposure during early life and the risk of MetS. However, these results are controversial, which may be for the reason of different study designs, races of participants, causes of famine, and diagnostic criteria. Therefore, we conducted this meta-analysis to summarize the effect of famine exposure during early life on MetS.

## 2. Materials and Methods

We referred to Preferred Reporting Items for Systematic Review and Meta-Analyses (PRISMA) guidelines to carry out this meta-analysis.

### 2.1. Search Strategy

We searched the PubMed, Web of Science, Embase, ScienceDirect, and Chinese National Knowledge Infrastructure up to December 2019 for articles that explored the association between famine exposure during early life and the MetS in adulthood. The keywords were searched as follows: “famine OR starvation OR hunger OR undernutrition OR malnutrition” AND “metabolic syndrome OR MetS.” In addition, we searched the reference lists of retrieved articles manually.

### 2.2. Inclusion Criteria

Studies were included when the following criteria were met: (1) published as original articles; (2) the exposure of interest was famine; (3) the outcome of interest was MetS; (4) odds ratios (ORs) or relative risks (RRs) or hazard ratios (HRs) with 95% confidence intervals (CIs) were available; and (5) the most recent study was selected if data from the same population had been published more than once.

Two authors reviewed titles and abstracts independently, followed by full texts, to assess eligibility for inclusion, and any disagreements were solved by discussion.

### 2.3. Data Extraction and Quality Assessment

We extracted following data regarding the characteristics of the studies: name of the first author, year of publication, study design, age range or mean age, definition of MetS, sample size and number of cases, famine duration periods, causes of famine, and covariates adjusted for in each study.

The quality of the studies was assessed by the Newcastle-Ottawa Scale (NOS) [18] on 3 aspects, including selection of participants, comparability of participant groups, and outcome assessment. The NOS is widely used for assessing the quality of observational studies, with the versions of cohort and case-control studies and an adaptation for cross-sectional studies [19].

### 2.4. Statistical Analysis

Statistical analysis was performed using the command meta-analysis in Stata 12. Pooled OR with 95% CI was determined to assess the strength of association between famine exposure and the risk of MetS. The *I*^2^ statistics and chi-squared test were used to estimate the heterogeneity between the studies. The *I*^2^ values of 0, 25, 50, and 75% represent no, low, moderate, and high heterogeneity, respectively [20], while the *P* value of chi‐square statistics < 0.05 represents significant heterogeneity. When *I*^2^ > 50% or *P* value of chi‐square statistics < 0.05, the random-effects model was used; otherwise, the fixed-effects model was used [20, 21]. Subgroup analyses were performed based on study design, gender, exposure type, definition of MetS, causes of famine, and adjustment status for age, smoking, drinking, and physical activity. Meta-regression was performed for further evaluation of the source of heterogeneity. Sensitivity analysis performed by sequential omission of individual studies was carried out to assess the stability of the results and the key study with substantial impact on the between-study heterogeneity [22]. Publication bias was evaluated by Begg's and Egger's tests and funnel plots.

## 3. Results

### 3.1. Literature Search

**Figure 1:**
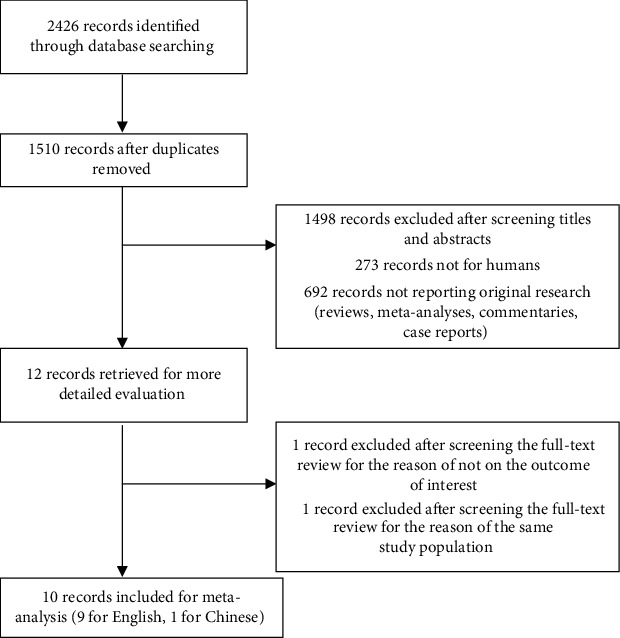
Flow chart of study selection.

A total of 1510 articles were retrieved based on the search strategy. 1498 potential articles were included after duplicates were removed, and 12 articles retrieved for more detailed evaluation after titles and abstracts were screened. Finally, 39 studies of 10 articles were included in the present meta-analysis (9 in English [23–31] and 1 in Chinese [32]). The flow chart is presented in Figure 1.

### 3.2. Study Characteristics and Quality Evaluation

There were 10 articles involving of 81504 participants from Europe and Asia evaluating the association between famine exposure and the risk of MetS in adulthood. The characteristics of the identified studies are presented in Table 1. These articles were published between 2007 and 2019, with the number of participants ranging from 783 to 25708. Among them, 2 articles were cohort studies, and 8 articles were cross-sectional studies. In terms of the definition of MetS, 4 articles used the United States National Cholesterol Education Program Adult Treatment group third guide (NCEP-ATPIII), 2 articles used the Chinese Diabetes Society in 2004 (CDS 2004) criteria, 3 articles used International Diabetes Foundation criteria (IDF), 2 articles used the Chinese Diabetes Society in 2013 (CDS 2013) criteria (the diagnostic criteria of 2017 version are the same as the 2013 version, and we recognized these criteria as the 2013 version), 1 article used a proxy variable based on the simultaneous occurrence of hypertension, obesity, diabetes (as a proxy for fasting hyperglycemia), and dyslipidemia (as a proxy for hypertriglyceridemia), and 1 article included in our research used the definition of NCEP-ATPIII, IDF, and CDS 2013 separately [31]. With regard to causes of famine, 3 articles were war and 7 articles were natural disaster. Among the 39 studies from the 10 articles, 16 studies showed a positive association, while 23 studies showed no significant association between them. Additionally, all articles were assessed for quality according to the NOS, and the qualities were ranged from moderate to good, scoring between 6 and 8. Tables 2 and 3 report the results of quality assessment.

### 3.3. Main Analysis

**Figure 2:**
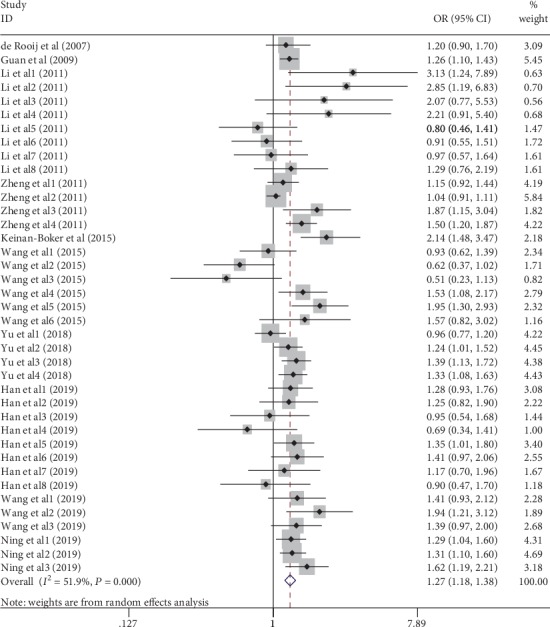
Forest plot of association between famine exposure and MetS.

The meta-analysis results of the 39 studies showed that famine exposure during early life was associated with a significantly increased risk of MetS (OR = 1.27, 95% CI: 1.18-1.38) (Figure 2), with moderate heterogeneity (*I*^2^ = 51.9%; *P*_heterogeneity_ < 0.001) (explanatory note: in Ning's study [31], the result suggested that the CDS definition is superior to the other definitions for determining the association between famine exposure and MetS, and the pooled OR combined the ORs of Ning's study in definition of CDS 2013). In subgroup analyses conducted by study design, definition of MetS, causes of famine, adjustment status for age, smoking, drinking, and physical activity, the results remained consistent. However, subgroup analyses conducted by gender, exposure type revealed no relation between famine exposure and the risk of MetS when pooling ORs of studies on male, adolescence/adult exposure type. The results of subgroup analyses are summarized in Table 4.

### 3.4. Metaregression

The *P* values from univariate metaregression with the covariates of study design, gender, exposure type, definition of MetS, causes of famine, adjustment status of age, smoking, drinking, and physical activity were 0.977, 0.318, 0.916, 0.531, 0.865, 0.859, 0.696, 0.676, and 0.871, respectively. The results showed that no covariate contributed a significant impact on between-study heterogeneity.

### 3.5. Sensitivity Analysis

The results suggested the association between famine exposure and MetS was stable. By omitting one study sequentially, the pooled ORs ranged from 1.22 (95% CI: 1.02-1.46) to 1.29 (95% CI: 1.06-1.57). To further find the potential source of between-study heterogeneity, we also used the leave-one-out method. No study was found to contribute to between-study heterogeneity.

### 3.6. Publication Bias

**Figure 3:**
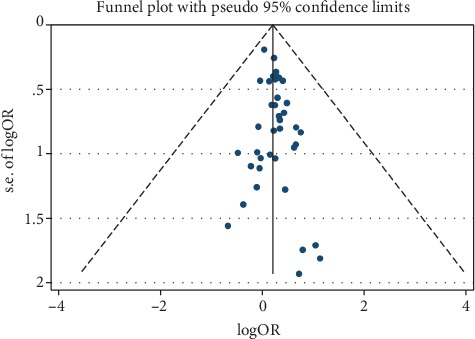
Funnel plot for the association between famine exposure and MetS.

No publication bias among the included studies was identified though the funnel plots, Begg's test (*P* = 0.981), and Egger's test (*P* = 0.150) (Figure 3).

## 4. Discussion

In recent decades, there has been an ongoing discussion about the relationship between the famine exposure during early life and the risk of MetS in adulthood, which has showed inconclusive results. To the best of our knowledge, this is the first meta-analysis exploring the relationship between famine exposure and the risk of MetS in adulthood. Current results combined ORs of 39 studies from 10 articles suggested that famine exposure was associated with an increased risk of MetS. Furtherly, the relationship remained consistent in subgroup analyses conducted by study design, definition of MetS, causes of famine, adjustment status for age, smoking, drinking, and physical activity. Thus, this meta-analysis may represent the best available evidence on the consistency and strength of the association between famine exposure during early life and the risk of MetS in adulthood. We think this finding is important for it provides a new approach for prevention of MetS in adulthood.

Subgroup analysis conducted by gender suggested that there was not a significant increase in MetS in men. Reasons might be male fetuses' and infants' susceptivity to adverse environmental conditions [33], which causes higher mortality rate. Furthermore, the survivors might be healthier than those who prematurely died. Another hypothesis of gender-specific difference to placental environment may also partially explain it. Female placentas adapt more quickly than male to an adverse intrauterine environment, leading to decreased growth without growth restriction. This is a key mechanism which allows females to survive but links early development with later-life disease [34, 35].

Subgroup analysis conducted by exposure type suggested that exposure to famine during fetal life and childhood was associated with higher risk of MetS. The following aspects may partially explain it. Firstly, catch-up growth may compensate it when undernutrition emerges during early life development. Interventional studies using animal models have reported that adaptive responses for stabilizing conceptus growth and enhancing postnatal fitness are related to undernutrition of preimplantational embryos, leading to postnatal metabolic disease [36]. Catch-up growth is also a mechanism that benefits the newborns' possibilities of survival, resulting in the growth of the vital organ like the brain at the expense of other organs like the kidney and pancreas, which could have adverse effects on adaptation to nutritional abundance in later life [37]. Studies have demonstrated that the catch-up growth was associated with the high blood pressure, insulin resistance, and obesity in adulthood [38–40]. Secondly, micronutrient deficiency during the fetal and childhood period may cause impaired organ development and oxidative stress [41–44] and result in grown-up chronic diseases furtherly. Finally, epigenetics is suggested to be the molecular mechanism linking early life famine exposure to growth and metabolism, such as DNA methylation and histone modification [45, 46], which may cause fetal programming of postnatal disease susceptibility.

A few studies have compared different diagnosis criteria for MetS applied in different populations [47–49]. Studies have found that the NCEP criterion has higher detection rates and that the CDS 2004 criterion has lower detection rates and lower compliance rates compared with other diagnosis criteria. The judgment criteria of obesity, high blood pressure, and high blood glucose by CDS 2004 are different from NCEP criterion and IDF criterion. For example, the determination of obesity, the CDS 2004 criterion is based on BMI, while other criteria are based on waist circumference. A recent study conducted in China has compared the CDS 2013 criterion with the NCEP criterion, the IDF criteria and the 2009 joint interim statement of the International Diabetes Federation Task Force on Epidemiology and Prevention (JIS) criteria among the elderly in Nanjing [50], which suggested that the CDS 2013 criterion also has a lower detection rate. In the CDS 2013 criterion, the cutoff points for high waist circumferences and high blood glucose are higher than other criteria, and no distinction between men and women in the standard of low high-density lipoprotein cholesterol (HDL-C) leads to a lower low HDL-C detection rate in women.

However, 1 article included in our research suggested that the CDS definition is superior to the other definitions for determining the association between famine exposure and MetS, because the results showed that adolescence/adult famine exposure was significantly associated with MetS risk only for the CDS definitions [31]. But in our meta-analysis, the result was consistent regardless of diagnosis criteria.

This meta-analysis has several potential limitations. Firstly, due to lack of available data from original articles, this meta-analysis could not analyze the severity of famine exposure and the risk of MetS. Secondly, most of the included studies were cross-sectional studies, which may suffer from bias and low comparability unavoidably. Thirdly, only published studies were included in this meta-analysis, so publication bias may have occurred despite no publication bias was identified from funnel plots, Begg's test, or Egger's test.

## 5. Conclusions

In conclusion, this meta-analysis indicates that famine exposure during early life is associated with an increased risk of MetS in adulthood. The result suggests that maternal nutritional state during gestation periods and children's nutritional state should be given particular attention. Interventions are needed to prevent undernutrition during early life for reducing the prevalence of MetS. Future research studies, especially well-designed cohort studies, are needed to confirm the conclusion.

## Figures and Tables

**Table 1:** Characteristics of studies included for famine exposure and the risk of metabolism syndrome.

First author, year	Continent	Study design	Age range Mean age (exposed/unexposed)	Definition of MetS	Study size (cases/participants)	Famine duration periods	Causes of famine	Adjustment for covariates
de Rooij, 2007	Europe	Cohort	58.0	NCEP-ATPIII	251/783	1944-1945	War	Gender
Guan, 2009	Asia	Cross-sectional	43-53	CDS 2004	1475/14917	1959-1961	Natural disaster	Age
Li, 2011	Asia	Cross-sectional	38-50	NCEP-ATPIII	646/7874	1959-1961	Natural disaster	Gender, family income, family history of diabetes and hypertension, educational level, current smoking, alcohol use, physical activity level, and BMI
Zheng, 2011	Asia	Cross-sectional	44-51	CDS 2004	756/5040	1959-1961	Natural disaster	Age
Keinan-Boker, 2015	Asia	Cross-sectional	69.4/69.2	Metabolic syndrome proxy	150/1086	1940-1945	War	Gender and age
Wang, 2015	Asia	Cross-sectional	40-55	IDF	NR^a^/6445	1959-1962	Natural disaster	Age, smoking, rural/urban residence, and economic status
Yu, 2018	Asia	Cohort	49-61	IDF	2396/7915	1959-1961	Natural disaster	Gender, education, smoking status, drinking status, physical activity, past history of CHD, family history of hypertension and diabetes, fruit intake, vegetable intake, meat intake, BMI, and famine severity
Han, 2019	Asia	Cross-sectional	53-80	NCEP-ATPIII	NR^a^/25708	1951-1953	War	Age, household income, smoking, drinking, and exercise status
Wang, 2019	Asia	Cross-sectional	51.8/54.0/55.5/48.9	CDS 2013	799/2148	1959-1961	Natural disaster	Gender, smoking status, drinking status, physical activity level, parents, and their own education level
Ning, 2019	Asia	Cross-sectional	51.3	CDS2013/NCEPATPIII/IDF	2809/9588	1959-1961	Natural disaster	Age, study cohort, residential area, sex, education levels, income levels, current smoking, and current drinking

^a^Not reported.

**Table 2:** Results of quality assessment for cross-sectional studies.

First author, year	Selection				Comparability	Outcome	
	Representativeness of the sample	Sample size	Nonrespondents	Ascertainment of the exposure	Based on the study design or analysis	Assessment of the outcome	Statistical test
Guan, 2009	+			+	+	++	+
Li, 2011	+			+	+	++	+
Zheng, 2011	+			+	+	++	+
Keinan-Boker, 2015	+		+	+	+	+	+
Wang, 2015	+		+	+	+	++	+
Han, 2019	+			+	+	++	+
Wang, 2019	+			+	+	++	+
Ning, 2019	+			+	+	++	+

**Table 3:** Results of quality assessment for cohort studies.

First author, year	Selection				Comparability	Outcome		
	Representativeness of exposed	Selection of nonexposed	Ascertainment of exposure	Outcome not present at start	Based on the study design or analysis	Ascertainment of outcome	Length of follow-up	Adequacy of follow-up
de Rooij, 2007	+	+	+		+	+	+	+
Yu, 2018	+	+	+		++	+	+	+

**Table 4:** Results of subgroup analysis for famine exposure and MetS risk.

Subgroup	No. of studies	OR (95% CI)	*I* ^2^ (%)	*P*_heterogeneity_
Study design				
Cross-sectional	34	1.29 (1.18-1.42)	54.4	<0.001
Cohort	5	1.22 (1.11-1.35)	40.0	0.154
Gender				
Male	9	1.04 (0.96-1.13)	32.7	0.156
Female	9	1.48 (1.31-1.67)	0	0.615
Male/female	21	1.32 (1.21-1.45)	39.9	0.031
Exposure type				
Fetal	14	1.27 (1.14-1.43)	48.2	0.023
Childhood	20	1.29 (1.16-1.44)	54.7	0.002
Adolescence/adult	5	1.03 (0.65-1.61)	65.9	0.020
Definition of MetS				
NCEP-ATPIII	20	1.24 (1.14-1.35)	6.0	0.382
CDS 2004	5	1.25 (1.07-1.47)	73.4	0.005
CDS 2013	6	1.39 (1.24-1.55)	0	0.594
IDF	13	1.21 (1.07-1.37)	56.0	0.007
Metabolic syndrome proxy	1	2.14 (1.48-3.47)	—	—
Causes of famine				
Natural disaster	29	1.28 (1.17-1.40)	58.4	<0.001
War	10	1.28 (1.13-1.46)	20.9	0.251
Adjust for age				
Yes	23	1.27 (1.15-1.40)	58.6	<0.001
No	16	1.29 (1.13-1.46)	40.9	0.046
Adjust for smoking				
Yes	32	1.15 (1.03-1.29)	42.7	0.006
No	7	1.31 (1.12-1.54)	73.5	0.001
Adjust for drinking				
Yes	26	1.28 (1.20-1.37)	24.8	0.125
No	13	1.27 (1.09-1.47)	72.2	<0.001
Adjust for physical activity				
Yes	23	1.25 (1.16-1.35)	28.3	0.102
No	16	1.29 (1.15-1.46)	68.7	<0.001
